# Designing Entrustable Professional Activities for Treatment Planning of Oral Cancer by Maxillofacial Surgery Residents: A Modified Delphi Study

**DOI:** 10.1155/2024/5516332

**Published:** 2024-09-14

**Authors:** Maidah Hanif, Yawar Hayat Khan, Kamran Ali

**Affiliations:** ^1^ Foundation University College of Dentistry and Hospital, Islamabad, Pakistan; ^2^ Riphah International University, Rawalpindi, Pakistan; ^3^ QU Health College of Dental Medicine, Doha, Qatar

## Abstract

**Purpose:**

The aim of this study was to develop a competency framework based on entrustable professional activities (EPAs) in oral cancer management by postgraduate trainees in oral and maxillofacial surgery through expert consensus.

**Materials and Methods:**

The study design was based on a modified Delphi technique and involved iterative online surveys with two rounds of data collection and analysis. Initial development of the questionnaire identified five EPAs based on 42 competencies along with supervision level and assessment strategies. The first Delphi round involved administration of the survey questionnaire online to maxillofacial surgeons meeting the inclusion criteria for experts. Consensus was achieved on five EPAs and 36 competencies (≥80% response rate). Six competencies were rephrased and sent again in the Round 2 questionnaire to achieve a consensus.

**Results:**

A total of 45 experts participated in Round 1 followed by input from 27 experts in Round 2 of the Delphi panel. Following two iterative rounds of online surveys and feedback, expert consensus was achieved to develop an EPA framework in five EPA domains focused on the management of oral cancer by postgraduate trainees in maxillofacial surgery including 38 specific competencies, supervision level, and assessment strategies. High content validity of the study was established through a comprehensive literature search, and expert feedback was evidenced by an excellent response rate (93.34%, and 64.28%) and a stringent criteria of response agreement amongst experts (≥80%).

**Conclusion:**

In conclusion, this study employed expert consensus to identify five EPAs with 38 competencies along with the required supervision level of postgraduate maxillofacial trainees for the management of oral cancer. This EPA framework provides a roadmap for training supervisors to map the learning outcomes in oral oncology for postgraduate trainees in oral and maxillofacial surgery.

## 1. Introduction

Cancer is currently one of the most common diseases responsible for premature mortality in most countries of the world and accounted for more than 10 million deaths in 2020. Oral cancer is the 11th most prevalent cancer globally [1, 2]. Oral squamous cell carcinoma (OSCC) is the most common type of malignancy in the oral cavity and accounts for over 90% of oral cancers. Other types of malignancies, such as sarcomas and lymphomas, are encountered infrequently in the oral cavity [3]. The Global Cancer Observatory data for 2020 shows that the annual incidence of OSCC was 377,713 cases worldwide with the highest number recorded in Asia (248,360), followed by Europe (65,279) and North America (27,469) [1]. The 5-year prevalence of OSCC approached nearly 1 million (959,248) and mirrored the same trends, i.e., highest incidence in Asia, followed by Europe and North America. Smoking, alcohol, exposure to DNA oncogenic viruses, betel nut use, and immunosuppression are recognized risk factors for oral cancer [4, 5].

Despite unprecedented advancements in the management of oral cancer in recent years, the survival rates of head and neck cancers have shown modest improvements [6]. Importantly, the burden of OSCC is expected to rise and the Global Cancer Observatory the incidence of OSCC is predicted to increase by up to 40% by 2040 with a corresponding increase in mortality [1, 2]. These trends in the global incidence of oral cancer underscore the importance of training the future healthcare professionals involved in the prevention and management of oral cancer to achieve improved patient outcomes.

Early recognition of oral cancer remains a fundamental goal to improve treatment outcomes. It is recognized that dentists and allied dental care professionals play a crucial role in the early recognition of oral cancer and urgent referral for treatment in specialist settings [7, 8]. Given that public screening of oral cancer on a mass scale is not viable, regular dental check-ups remain a suitable option for opportunistic oral cancer screening. Routine use of visual oral screening may reduce oral cancer mortality among the high-risk groups, such as users of tobacco and alcohol [7, 9].

In most developed countries, meticulous oral examination for identification of suspicious oral precancerous and cancerous lesions is part of a dental professionals' duty of care. Dental professionals are also expected to provide preventive advice regarding lifestyle risk factors such as smoking, alcohol, and betel nut consumption [8]. Moreover, there is growing recognition of the need for public health education to raise awareness regarding the risk of UADT and oral cancers caused by human papilloma virus [9, 10]. Evidence from the literature suggests that dental professionals need additional education and training to provide effective patient education on the risks of oral cancer due to human papilloma virus infection which is often related to sexual practices and requires sensitive communication [11].

In contemporary healthcare, oral cancer patients are managed by a multidisciplinary team (MDT) and are aimed at providing the most appropriate management of oral cancer patients with input from a range of specialists [10]. The MDT for oral cancer includes oral and maxillofacial surgeons, ear nose and throat (ENT) surgeons, plastic and reconstructive surgeons, along with experts in radiology, pathology, oncology, anesthesia, speech and language therapists, dieticians, nursing, physiotherapists, prosthodontists, and social workers. This approach has shown significant benefits to improve evidence-based treatment planning of oral cancer patients and a positive impact on the quality of life during and after cancer treatment [10, 11].

Oral cancer management is a core component of specialist training programs in maxillofacial surgery. Given the growing incidence and prevalence of oral cancer globally, it is imperative that future maxillofacial surgeons receive structured, competence-based training in the prevention and management of oral cancer. Although oral cancer recognition and management are included in the curricula of postgraduate training programs in maxillofacial surgery, there are considerable variations in the degree of clinical exposure and experience gained by oral and maxillofacial trainees globally. The training programs should aim to encompass the clinical and technical developments in oral oncology practice and also impart training to address the physical and social needs of oral cancer patients [12, 13].

Given the challenges in providing structured training in the management of oral cancer to oral and maxillofacial surgery trainees, there is a need to achieve a consensus on the clinical skills and competencies to improve patient outcomes. The providers of oral and maxillofacial training programs may consider novel frameworks to review their existing curricula and redefine the competencies and professional activities in oral cancer management expected from oral and maxillofacial trainees on completion of their training programs. Entrustable professional activities (EPAs), a relatively new competency-based assessment framework, links competencies to practice and provides relevant assessments of student capacity and performance. Introduced by Ten Cate in 2005, the framework provides a “mode of translation of competencies into clinical practice” by combining multiple competencies to formulate entrustable clinical activities a competent clinician could be entrusted to perform [14]. EPAs are measurable, observable clinical tasks that may be taught, evaluated, and then entrusted as a learner reaches a certain level of competence [15, 16]. EPAs are being used increasingly to define the activities of healthcare training programs in medicine, dentistry, nursing, and pharmacy [15, 17, 18, 19, 20, 21, 22]. Appropriately designed EPAs encompass several domains of competency, include several competencies in each EPA, and are mapped to specific milestones during a training program [23].

The use of an EPA framework in oral and maxillofacial surgery training programs can identify developmental activities which the residents can be expected to carry out to develop competence in treatment planning of head and neck oncology. The framework for EPAs identifies the level of supervision required by the trainees at various stages of the program and can facilitate the assessment of trainees' performance to make informed decisions regarding the abilities of the trainees to perform these tasks independently without supervision [16]. Defining the educational objectives through the lens of EPAs can guide both the trainees and their supervisors to develop a roadmap for effective postgraduate oral and maxillofacial surgery education.

The aim of this study was to develop a competency framework based on EPAs in oral cancer management by postgraduate trainees in oral and maxillofacial surgery through expert consensus.

## 2. Materials and Methods

### 2.1. Research Ethics

This research was conducted in accordance with the Declaration of Helsinki ethical principles for medical research involving human subjects, including research on identifiable human material and data. Ethical approval was obtained from the ethical review committee of Riphah International University, Islamabad, Pakistan (Reference no. Riphah/IIMC/ERC/IRC/22/2009). Participation in the study was voluntary and all participants were required to provide an informed consent prior to their participation in the study. All data were collected, analyzed, and reported anonymously.

### 2.2. Study Design

The study design was based on a modified Delphi technique and involved iterative online surveys with two rounds of data collection and analysis. Following the development of the questionnaire, the first Delphi round involved administration of the survey questionnaire online. Data from the first round were collected and analyzed to identify common themes and areas of agreement and disagreement. The questionnaire was refined based on responses of the participants and data analyses. The updated questionnaire was shared with the participants in the second Delphi round to achieve a consensus. This technique allowed adaptive modification of the EPA framework allowing modifications at each stage of the study.

### 2.3. Settings

The study was conducted online and the participants were expected to provide their responses to online surveys in two iterative online surveys

### 2.4. Study Duration

The study was conducted over 6 months from January 2022 to June 2022.

### 2.5. Selection of Delphi Panelists

The Delphi panel was constituted using a predefined inclusion criteria and all participants were required to meet the following requirements:

- A specialist qualification in oral and maxillofacial surgery.
- Valid registration with the national regulatory body in the country of practice.
- At least 5 years' experience as a consultant oral and maxillofacial surgeon in a public or private hospital.

### 2.6. Sampling Technique

A convenience sampling technique was employed to recruit national and international experts in oral and maxillofacial surgery in South East Asia, Middle East, and Europe using the professional channels of the research team. Potential participants were invited to participate in the Delphi panel through WhatsApp or email by the research team. The invites were accompanied by a participant information sheet explaining the aims and scope of the study and what the participants were expected to do. All participants were required to sign an informed consent to confirm their participation in the panel. The study aimed to recruit a minimum of 15 experts for the Delphi panel based on the guidelines in the literature [24, 25].

### 2.7. Data Collection

**Figure 1:**
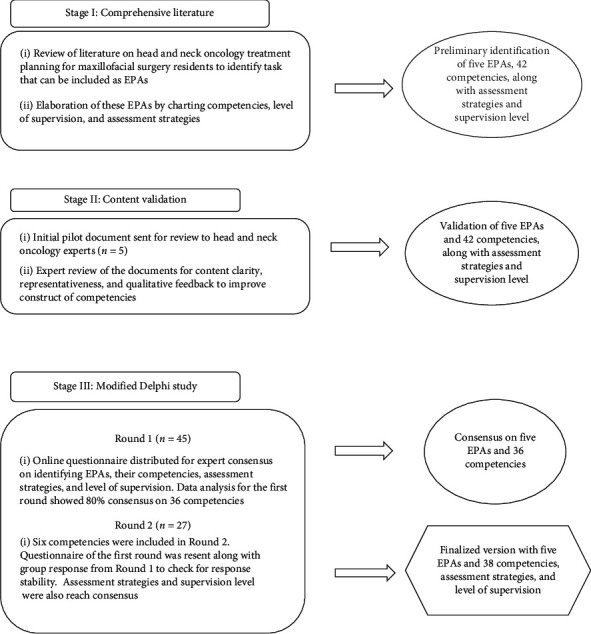
Stages of data collection.

The study was conducted in three stages as depicted in Figure 1.

#### 2.7.1. Stage I—Literature Review and Questionnaire Development

In-depth literature searches to identify a variety of competencies and assessment strategies for the postgraduate oral and maxillofacial surgery residency program for head and neck oncology treatment planning. Different national and international undergraduate curricula as well as literature on learning outcomes and competencies in postgraduate oral and maxillofacial surgery residency were sought, including PMDC, CPSP curriculum, GMC (General Medical Council) document for oral and maxillofacial surgery curriculum, International Association of Oral and Maxillofacial Surgery Guidelines for maxillofacial surgery residency, curriculum of European Association of Cranio-Maxillofacial Surgery, and curriculum of University of Minnesota on oral and maxillofacial surgery residency.

A questionnaire was prepared in which 42 competencies identified from the literature were grouped under five meaningful tasks (EPAs) for management of oral cancer patients by postgraduate trainees in maxillofacial surgery to prepare students who perform these EPAs under indirect supervision by the end of their last year of training. Assessment strategies for each EPA were also identified.

#### 2.7.2. Stage II—Content Validation

Prior to starting the main project, a pilot study was carried out and five consultant maxillofacial surgeons were selected using convenience sampling. The objective was to establish the face validity of EPAs and evaluate if they were specific, understandable, and relevant. The consultants found the questionnaire clear and understandable. Content validity measures how well items correspond or reflect a specific domain and is measured using qualitative techniques.

#### 2.7.3. Stage III—Modified Delphi Round

*(1) Delphi Round 1*. The participants were asked to assess and comment on the importance of the identified competencies in head and neck oncology treatment planning for maxillofacial surgery residency. Survey response time is 4 weeks. Five Likert points were used for each of the EPAs and their competencies, and participants were asked to rate each statement by level of importance. For each question, participants were invited to leave a comment box with any additional EPAs, competencies, assessment methods, or suggestions/feedback. Participants were also told how long it would take them to complete the survey (20–30 min). No new EPAs were identified in the second round. However, suggestions for more clarification of a few competencies were taken into account.

*(2) Delphi Round 2*. Following the first round's data analysis, a second questionnaire was created with questions that failed to garner consensus in Round 1. Forty-two people who replied in the first round received it through email link. Both the individual responses from Round 1 and the anonymized group responses were given to the participants. Additionally, they received the outcomes of the Round 1 consensus-based items. Once more the survey period ran for 4 weeks again (10–15 min is the time required to fill the questionnaire).

The participants were instructed to reconsider the items that failed to get consensus in Round 1. The same five-point Likert scale for level of importance was used. They were also requested to provide a justification if they changed their earlier responses or decided to reject the group consensus. For supervision level, ≥80% agreement on a specific level was set as criterion for inclusion.

### 2.8. Data Analyses

Descriptive statistical analysis of quantitative data was done utilizing SPSS version 28 (Statistical Package for Social Scientists by IBM Corp., New York, USA). The percentage responses, median, and interquartile ranges were calculated as well as the mean and standard deviation. The percentage ranking of evaluation strategies was also computed. The consensus criteria were defined as items with 80% or more agreement, median of ≥4 on a five-point Likert scale, and interquartile range ≤1. Qualitative data (open-ended responses in the comment boxes) were also analyzed. Items not reaching consensus in the first round were sent in the second round for reconsideration. In the last round, the list of EPAs with their competencies and assessment strategies was redistributed to experts for final approval and also to see the stability of responses. Reliability was computed for all rounds using Cronbach's alpha coefficient. Internal validity was ensured through an extensive literature search, involving substantial number of knowledgeable experts, the use of successive rounds, and a high agreement criterion for consensus.

## 3. Results

### 3.1. Round 1

The questionnaire with five EPAs, their 42 competencies were sent to 45 consultants oral and maxillofacial surgeons meeting inclusion criteria. Forty-two of them participated by completing the first-round questionnaire. Experts included national and international oral and maxillofacial surgeons meeting the inclusion criteria.

Experts graded the competencies for their importance on a five-point Likert scale from “very important to not at all important.” As per consensus criteria already defined, items with 80% or more consensus as extremely or especially important, with median of ≥4 and interquartile range ≤1 were included. Items not fulfilling this consensus criterion (four EPAs and six competencies) were sent in Round 2 questionnaire for reconsideration. The percentage agreement for these six competencies ranges from 4.8% to 71.4%. As per consensus criteria already defined, items with 80% or more consensus, median of ≥4 and interquartile range ≤1 were included. Only four items showed inconsistency with *p* value and were removed from the final EPAs after the second round.

### 3.2. Round 2

Four EPAs (with six competencies) did not meet consensus for minimum supervision level in Round 1. They were sent in Round 2 for reconsideration along with six competencies that did not meet the criteria for inclusion. Few statements were elaborated, and EPAs were rephrased after feedback from the first round. The Round 2 questionnaire was sent to 42 respondents who participated and completed Round 1. The consensus criterion for inclusion was the same as of Round 1. Only 27 participants responded for Round 2. At the end of Round 2, a set of five EPAs with 38 competencies (Tables A1–A11 of Appendices A and B) along with respective level of supervision and assessment strategies was identified after expert consensus (Table 1).

Experts were required to mark the relevant assessment strategies for each EPA, for percentage ranking and the level of supervision for each EPA. They could choose more than one assessment option, as shown in Table 2. For supervision level, the criterion was an agreement response of 80% or more.

By employing a sizable number of consultants in an expert panel with subject–matter expertise, a high response rate of the experts, the use of subsequent rounds, and a high standard of answer agreement, i.e., 80% or more, the study's high content validity was ensured. The study had a high-reliability coefficient depicting internal consistency. Moreover, the McNemar change test showed stability of responses at the end of second round, as shown in Table 3.

## 4. Discussion

The modified Delphi technique used in this study was used to engage maxillofacial consultants to establish desired EPAs for treatment planning in head and neck oncology, their competencies, and assessment strategies at Level 4 supervision. For postgraduate residents in maxillofacial practice, these EPAs represent essential tasks which must be undertaken prior to completion of a training program. Consensus techniques in medical education research offer a useful way to integrate the views of relevant professional experts to support decision-making. The Delphi process was employed as it is considered the most practical and rigorous method to achieve consensus among geographically dispersed experts as it is based on the premise that pooled intelligence enhances individual judgment and captures the collective opinion of a group of experts without being physically assembled [25]. Studies show that the Delphi method shows superior accuracy to other expert judgment methods like traditional group discussions, conferences, and other interactive group processes [26]. The modified Delphi was chosen as the study design considering the aim to develop a systematic, consensus-based, assessment framework for maxillofacial residency, EPAs, and integrating the desired competencies [27]. The current research employed a modified Delphi study as the first round was a structured questionnaire rather than a pure qualitative round as in classic Delphi. Comment boxes were provided with every question for any suggestions. Time limitations and the limited familiarity of the participants with EPAs framework were recognized and, therefore, a predefined set of EPAs based on competencies postgraduate oral and maxillofacial curricula was used.

Previously published studies have used Delphi/modified Delphi technique to develop specialty-specific EPAs including anesthesia, neonatology, and internal medicine; however, they focused on the initial step of identifying the core list of EPAs [17, 18, 19, 22, 27]. In the current study, a consensus was achieved not only on the final list of EPAs but on their respective set of competencies and assessment strategies. Also, the stability of responses was evaluated at the end of the second round.

Many Delphi studies use certain levels of agreement and/or descriptive statistics (mean/median and standard deviation/interquartile range) to quantify consensus amongst an expert panel [28]. In the current study, three types of measurements including percentage agreement, median, and interquartile range were used, applying the following criteria for consensus for inclusion; ≥80% participants' agreement in the Top 2 rating (extremely and very important), median score ≥4, and interquartile range ≤1 on five-point Likert scale in Rounds 1 and 2. Statistical analysis of Round 1 revealed that three EPAs and six competencies did not meet the criterion and were resend in Round 2. One of the key elements of the Delphi process is to provide group responses and participants' individual response after every round [25]. The purpose is to provide an opportunity for experts to reconsider their opinion based on the group response, if desired. For the second questionnaire, the criteria for dropping out any EPA or competency that was rated 1 and 2 (not at all/slightly important) on a five-point Likert scale was made. One EPAs met this criterion, and their competencies were deleted from the final questionnaire of Round 2. The Delphi technique aims to produce consensual and consistent opinions from experts over iterative rounds. Response stability was assessed by computing *p* values using McNemar change test. The study was concluded once the consensus was achieved at the end of the second round.

Although a Delphi technique is considered suitable to map the learning outcomes of a program into an EPA framework, the professional background of the panelists poses a risk of bias. This was addressed through the inclusion of panelists from diverse backgrounds, maintaining anonymity of the panelists, providing structured feedback, defining clear consensus criteria, using neutral facilitators, and applying rigorous data analysis techniques. The panelists included experts from diverse geographic locations in South East Asia, Middle East, and Europe with varying levels of experience ranging from 5 to 30 years. Anonymity of the panelists was maintained throughout the Delphi panel proceedings as the participants provided their individual responses remotely using online surveys. Anonymity of the participants was also maintained in data processing and reporting and it is not possible to identify individual members of the Delphi panel from the data reported in the manuscript. As participants were not aware of other members of the Delphi panelists, it helped to prevent domination of individual views and perspectives, and consensus was achieved with inclusion of a diverse group of experts [25]. The methodological rigor was maintained with a high response rate at each stage. The participants were followed up and reminder emails and WhatsApp texts were sent to ensure a high response rate. The response rate was 93.34% for Round 1 and 64.28% for Round 2. The iterative rounds of Delphi are useful to establish the content validity. The validity was enhanced by the involvement of substantial number of national and international consultants, high response rates, the use of successive rounds, and a high criteria of response agreement, i.e., 80% or more [28]. The Delphi process enhances reliability owing to anonymity of participants to each other in decision-making process, eliminating bias. Large panel size and iterative rounds also increase reliability [29]. Cronbach's alpha was calculated for internal consistency of the questionnaire in all rounds. The number of items and their inter-relatedness affects the value of alpha. Cronbach's alpha values range from 0 to 1, with a score of 0.7 or higher acceptable [30].

This study is the first to view the competencies in the management of oral cancer through the lens of EPAs and provides an integrated, holistic competency-based assessment framework which can be used to translate abstract competencies into clinical practice [16]. Given that the postgraduate students are expected to perform clinical activities under supervision, all EPAs for oral and maxillofacial residency are aimed at supervision Level 4, adopting the revised entrustment scale for postgraduate medical education [31, 32]. These EPAs encompass essential components in most postgraduate maxillofacial training curricula for head and neck oncology. The EPAs for oral and maxillofacial surgery residency in treatment planning of head and neck oncology identify observable, measurable activities that can be linked to existing residency objectives. They can provide greater clarity to existing objectives aiding effective training and assessment. Each EPA for treatment planning of oral cancer in maxillofacial surgery residency includes recommendations for assessment methods as well as the expected competencies in terms of knowledge, skills, and attitudes. The EPAs identified from the study can also serve as a self-assessment tool for medical and dental hospitals to improve their existing postgraduate oral and maxillofacial surgery training and assessment.

Predictive models by the Global Cancer Observatory show that unfortunately oral cancer is here to stay for many decades to come and therefore it is important that the fight against oral cancer must continue [33]. The importance of improving public awareness regarding risk factors for oral cancer, and a focus on oral health maintenance cannot be overstated [34, 35, 36]. Concerted efforts from national and global policymakers and professional bodies are required to reduce the global burden of oral cancer including a renewed focus on prevention [37]. Given the rapid developments in the field of oral cancer, it is inevitable that the technological advancements such as artificial intelligence, identification of cancer biomarkers, and the use of live imaging techniques will impact heavily on the diagnosis and management of oral cancer [38]. Therefore, like all educational curricula, EPA frameworks are dynamic and should evolve in the light of scientific developments and evidence-based clinical practices [23].

The main limitation of this study is that the methodology was focused on establishing content validity. It is imperative that the proposed EPA framework is tested in multiple training centers to evaluate its feasibility and utility in clinical educational settings and how it integrates with other workplace-based assessment tools. Feedback from educational providers, trainees, and patients can be used to refine and develop the proposed EPA framework further.

In conclusion, this study employed expert consensus to identify five EPAs with 38 competencies along with the required supervision level of postgraduate oral and maxillofacial trainees for treatment planning in oral oncology. This EPA framework provides a roadmap for training supervisors to map the learning outcomes in oral oncology for oral and maxillofacial surgery trainees.

## Figures and Tables

**Table 1:** Supervision level for EPA.

S. no	EPAs	Level 1 (%)	Level 2 (%)	Level 3 (%)	Level 4 (%)	Level 5 (%)
(1)	EPA 1 Diagnosis and treatment planning of patients with squamous cell carcinoma of the oral cavity	0.0	0.0	22.2	70.4	7.4
(2)	EPA 2 Diagnosis and treatment planning of skin cancers with basal cell carcinoma	0.0	0.0	18.5	70.4	11.1
(3)	EPA 3 Diagnosis and treatment planning of patients with salivary gland malignancy	0.0	22.2	3.7	74.1	0.0
(4)	EPA 4 Diagnosis, prognosis, and treatment planning of nonepithelial cancers of oral cavity	0.0	0.0	18.5	66.7	14.8
(5)	EPA 5 Presenting oral cancer cases in multidisciplinary team (MDT) meetings	0.0	0.0	0.0	40.7	59.3

**Table 2:** Percentage ranking of assessment strategies for EPAs.

EPAs	EMQS (%)	MCQ (%)	SEQ (%)	TOACS (%)	VIVA (%)	DOPS (%)	PORTFOLIO (%)	OSCE (%)	360° feedback (%)	OSTE (%)
EPA 1	2.0	19.4	24.5	16.3	16.3	6.1	8.2	4.1	2.0	1.0
EPA 2	9.2	15.6	19.3	15.6	11.9	6.4	5.5	11.9	2.8	1.8
EPA 3	5.2	16.7	22.9	20.8	10.4	5.2	7.3	9.4	1.0	1.0
EPA 4	2.1	17.9	24.2	9.5	17.9	5.3	12.6	5.3	3.2	2.1
EPA 5	2.4	7.1	11.8	17.6	17.6	10.6	9.4	5.9	10.6	7.1

**Table 3:** Reliability analysis and response rate.

Phase	Reliability (Cronbach's alpha)	Number of items (*N*)	Response rate (%)
Round 1	0.959	99	93.34
Round 2	0.856	99	64.28

**Table 4:** Questionnaire for Delphi round 1—EPA 1.

Number	Competencies	Very important (5)	Important (4)	Moderately important (3)	Slightly important (2)	Not at all important (1)
EPA 1: Diagnosis and treatment planning of patients with squamous cell carcinoma of the oral cavity						
Competencies related to knowledge						
	Identify all variants of squamous cell carcinoma related to the head and neck region					
(i)	Assess the overall medical condition of the patient including complete history, current condition, comorbidities, socioeconomic status of the patient, habits, past surgical procedures, medications, allergies, family history, and past treatment for the disease					
(ii)	Recognize, interpret, and communicate results of common pathologies on radiographic investigations (OPG X-ray, CT scan, and MRI)					
(iii)	Enumerate the features of metastatic cervical lymph node involvement on ultrasound or CT scan					
(iv)	Recognize the stage and grade of the disease, high-risk areas of the oral cavity and metastasis to other parts of the body					
(v)	Discuss the principles of different oncologic treatment modalities, including surgery, radiation therapy, chemotherapy, and immunotherapy					
(vi)	Discuss the principles of laser microsurgical resection, cryotherapy, robotic surgery, and/or electrosurgery					
(vii)	Identify predisposing factors pertaining to individual cases of head and neck neoplasm					
(viii)	Identify the principles of therapeutic and palliative radiation					
Competencies related to skills						
(i)	Perform clinical examination carefully and responsibly					
(ii)	Perform biopsy of the suspected lesion					
(iii)	Recognize, interpret, and communicate results of common histopathological variants of head and neck cancer					
(iv)	Document the history, clinical examination, and informed consent appropriately in the patient's healthcare record file					
(v)	Recognize when strong emotions (such as, anger, fear, anxiety, or sadness) are impacting an interaction and respond appropriately					
(vi)	Provide information on diagnosis and prognosis in a clear, compassionate, respectful, and objective manner					
(vii)	Recognize, value, and utilize the expertise of interprofessional team members					
(viii)	Understand steps of surgical procedure, potential risks, and means to avoid/overcome them					
(ix)	Efficiently performs surgical steps, avoiding pitfalls, and respecting soft tissue					
(x)	Perform surgical resection with clear margins (soft tissue/bone)					
(xi)	Perform neck dissection					
(xii)	Recognize and perform reconstruction with grafts, local, or free flaps					
Competencies related to attitude						
(i)	Show respectfulness to patients and family members					
(ii)	Be straightforward and kind					
(iii)	Promote a safe environment for the patient during the clinical examination					
	Respond to queries with understanding and patience					
(iv)	Appreciate patient's opinions, concerns, and apprehensions					
(v)	Communicate clearly and effectively with the patient and families regarding the disease					

**Table 5:** Questionnaire for Delphi round 1—EPA 2.

EPA 2: Diagnosis and treatment planning of skin cancers of face including lips						
Competencies related to knowledge						
(i)	Identify the most common malignant skin conditions					
(ii)	Determine the principles and techniques of facial reconstruction, including local and regional flaps and grafts					
(iii)	Identify the principles of laser microsurgical resection, cryotherapy, robotic surgery, and/or electrosurgery					
(iv)	Enumerate principles and techniques of frozen section diagnosis and Mohs micrographic surgery					
(v)	Identify the principles governing the use of local and systemic chemo- and immunotherapeutic agents					
(vi)	Identify the principles of therapeutic radiation					
Competencies related to skills						
(i)	Obtain informed consent for complex surgical procedures and therapies					
(ii)	Facilitate discussions with the patient and family in a way that is respectful, nonjudgmental, and culturally safe					
(iii)	Explain increasingly complex information in a manner comprehensible to the patient and family members					
(iv)	Resection of early skin cancer with clear margins					
(v)	Reconstruction with graft or local flap					
Competencies related to attitude						
(i)	Convey information related to the patient's health status, care, and needs in a timely, honest, and transparent manner					
(ii)	Exhibit appropriate professional behaviors and relationships in all aspects of practice, reflecting honesty, integrity, humility, commitment, compassion, respect, respect for diversity, and maintenance of confidentiality					
(iii)	Appreciate patient's opinions, concerns, and apprehensions					

**Table 6:** Questionnaire for Delphi round 1—EPA 3.

EPA 3: Diagnosis and treatment planning of patients with salivary gland malignancy						
Competencies related to knowledge						
(i)	Identify normal anatomy of the parotid, submandibular, and other salivary glands					
(ii)	Identify all malignant conditions related to the salivary glands					
(iii)	Enlist baseline laboratory investigations for patients requiring salivary gland malignant tumor management					
(iv)	Identify the facial nerve involvement, prerequisite test to confirm the involvement of the nerve					
(v)	Recognize and interpret abdominal and pelvis ultrasound and chest X-ray and other metastatic work workups before the surgery					
(vi)	Recognize the staging and grading of the malignant tumor of the head and neck area					
(vii)	Recognize the importance and levels of neck dissection for these patients					
Competencies related to skills						
(i)	Allocate enough time to patients in order to understand the importance of advised investigations					
(ii)	Identify normal anatomy on CT scan and MRI for parotid gland-related conditions as well as the pathological diseases					
(iii)	Perform clinical examination carefully and responsibly					
(iv)	Perform FNA/biopsy of the suspected lesion					
(v)	Document the history, clinical examination, and informed consent appropriately in the patient's healthcare record file					
(vi)	Understand steps of surgical procedure, potential risks, and means to avoid/overcome them					
(vii)	Employ a systemic approach to interpreting imaging findings on CT scans and MRI					
Competencies related to attitude						
(i)	Communicate clearly and effectively with the patient and families regarding the investigations					
(ii)	Communicate effectively and timely with fellow healthcare providers					
(iii)	Respond to queries with understanding and patience					
(iv)	Appreciate patient's opinions, concerns, and apprehensions regarding investigations					
(v)	Promote a safe environment for the patient during the radiological investigations					
(vi)	Recognize any limitations for any imaging modality and seek help as needed			\		

**Table 7:** Questionnaire for Delphi round 1—EPA 4.

EPA 4: Diagnosis, prognosis, and treatment planning of nonepithelial cancers of oral cavity						
Competencies related to knowledge						
(i)	Identify the sarcomas related to the head and neck region along with the most frequent sites					
(ii)	Recognize the metastatic workup of the disease					
(iii)	Recognize the staging and grading of the sarcoma of the head and neck region					
(iv)	Recognize and discuss the treatment and alternate options					
(v)	Recognize and explain the chances of success/failure and prognosis					
(vi)	Determine and explain the nature of the procedure intended to be performed and reconstruction options					
(vii)	Recognize and explain the nature of the procedure for getting clear margins by performing an intraoperative frozen section					
(viii)	Recognize and discuss the postoperative course					
(ix)	Discuss the nonsurgical treatment modalities in the neoadjuvant and adjuvant settings					
Competencies related to skills						
(i)	Elicit a history, perform a physical exam, select appropriate investigations, and interpret their results					
(ii)	Integrate all sources of information to develop a procedural or therapeutic plan that is safe, patient-centered, and considers the risks and benefits of all approaches					
(iii)	Obtain informed consent by discussing the indications, risks, benefits, and potential complications of the procedure planned with the patient and families					
(iv)	Triage a procedure or therapy, taking into account clinical urgency, the potential for deterioration, and available resources					
(v)	Efficiently performs surgical steps, avoiding pitfalls, respecting soft tissues, and getting clear margins					
(vi)	Map out an appropriate complete postprocedure plan					
(vii)	Discuss the disease and breaking bad news to the patient and family					
Competencies related to attitude						
(i)	Communicate clearly and effectively with the patient and families regarding the disease					
(ii)	Understand patient's expectations with the treatment plan					
(iii)	Respond to queries with understanding and patience					
(iv)	Encourage the patient and family to ask questions related to the treatment plan					

**Table 8:** Questionnaire for Delphi round 1—EPA 5.

EPA 5: Presenting oral cancer cases in multidisciplinary team (MDT) meetings						
Competencies related to knowledge						
(i)	Recognize and explain the team member's role in the procedure					
(ii)	Recognize and interpret the importance of a multidisciplinary team approach. (Oral and maxillofacial surgeon/head and neck surgeon, ENT, plastic surgeon, oncologist, prosthodontics, histopathologist, radiologist, rehabilitation team, nutritionist, and pain control team)					
(iii)	Correlate clinical presentation with the radiological findings of the disease					
(iv)	Enlist the effectiveness of neoadjuvant chemotherapy					
(v)	Enlist the indications, timing, and risk of adjuvant radiotherapy					
(vi)	Describe absolute and relative contraindications to the surgery					
Competencies related to skill						
(i)	Demonstrate understanding and kindness toward all team members					
(ii)	Formulate a justified request for an imaging examination to the team member					
(iii)	Document the MDT discussion in the healthcare record of the patient					
(iv)	Allocate enough time for the discussion of the patient and investigations with the team members					
Competencies related to attitude						
(i)	Show respectfulness to all team members					
(ii)	Maintain a dignified and respectful environment					
(iii)	Encourage all team members to ask questions related to the treatment plan					
(iv)	Communicate clearly and effectively with the patient and families regarding the MDT decision					
(v)	Recognize any limitations of the treatment and concerns of the team members					

**Table 9:** Questionnaire for Delphi round 2—EPA 1.

S. no.	Competencies	Very important (5)	Important (4)	Moderately important (3)	Slightly important (2)	Not at all important (1)
EPA 1: Diagnosis and treatment planning of patients with squamous cell carcinoma of the oral cavity						
Competencies related to knowledge						
(i)	Recognize, interpret, and communicate results of common pathologies on radiographic investigations (OPG X-ray, CT scan, and MRI) consensus not reached					

**Table 10:** Questionnaire for Delphi round 2—EPA 2.

EPA 2: Diagnosis and treatment planning of skin cancers of face including lips						
Competencies related to knowledge						
(i)	Identify the principles of laser microsurgical resection, cryotherapy, robotic surgery, and/or electrosurgery *Rephrased* (Identify the use of laser microsurgical resection and cryotherapy for the cutaneous lesions of the head and neck region)					
(ii)	Enumerate principles and techniques of frozen section diagnosis and Mohs micrographic surgery *Rephrased* (Identify the principles used for frozen section diagnosis of malignant skin lesions involving the head and neck region along with the importance of Mohs microscopic surgery in these conditions)					
(iii)	Identify the principles of therapeutic radiation (*Consensus not reached*)					

**Table 11:** Questionnaire for Delphi round 2—EPA 4.

EPA 4: Diagnosis, prognosis, and treatment planning of nonepithelial cancers of oral cavity						
Competencies related to knowledge						
(i)	Map out an appropriate complete postprocedure plan *Rephrased* (Outline an applicable treatment plan for the patient along with the postprocedure plan)					

**Table 12:** Questionnaire for Delphi round 2—EPA 5.

EPA 5: Presenting oral cancer cases in multidisciplinary team (MDT) meetings						
Competencies related to skill						
(i)	Document the MDT discussion in the healthcare record of the patient *Rephrased* (Documentation of the MDT discussion of the panel in the patient's healthcare record as well as in the file handed over to the patient for his/her own record)					

**Table 13:** Proposed assessment strategies for EPAs.

Number	EPAs	Assessment strategies									
		MCQs	SEQs	EMQs	TOCAS	Viva	OSCE	OSTE	DOPS	360° feedback	Portfolio
(1)	Diagnosis and treatment planning of patients with squamous cell carcinoma of the oral cavity										
(2)	Diagnosis and treatment planning of skin cancers of face including lips										
(3)	Diagnosis and treatment planning of patients with salivary gland malignancy										
(4)	Diagnosis, prognosis, and treatment planning of nonepithelial cancers of oral cavity										
(5)	Presenting oral cancer cases in multidisciplinary team (MDT) meetings										

**Table 14:** Proposed supervision level for EPAs.

S. no.	EPAs	Level of supervision				
		Level 1	Level 2	Level 3	Level 4	Level 5
(1)	Diagnosis and treatment planning of patients with squamous cell carcinoma of the oral cavity					
(2)	Diagnosis and treatment planning of skin cancers of face including lips					
(3)	Diagnosis and treatment planning of patients with salivary gland malignancy					
(4)	Diagnosis, prognosis, and treatment planning of nonepithelial cancers of oral cavity					
(5)	Presenting oral cancer cases in multidisciplinary team (MDT) meetings					

## Data Availability

The data underlying this article will be shared upon reasonable request from the corresponding author.

## Appendix

### A. Questionnaire: Round 1

- Any suggestions to improve/rephrase EPA.

____________________________________________

(ii) Kindly mention any other competencies you may like to include for this EPA.

____________________________________________

- Any suggestions to improve/rephrase EPAs.

____________________________________________

(ii) Kindly mention any other competencies you may like to include for this EPA.

____________________________________________

- Any suggestions to improve/rephrase EPA.

____________________________________________

(ii) Kindly mention any other competencies you may like to include for this EPA.

____________________________________________

- Any suggestions to improve/rephrase EPA.

____________________________________________

(ii) Kindly mention any other competencies you may like to include for this EPA.

____________________________________________

- Any suggestions to improve/rephrase EPA.

____________________________________________

(ii) Kindly mention any other competencies you may like to include for this EPA.

____________________________________________

### B. Questionnaire: Round 2

- Any suggestions to improve/rephrase EPA.

____________________________________________

(ii) Kindly mention any other competencies you may like to include for this EPA.

____________________________________________

- Any suggestions to improve/rephrase EPAs.

____________________________________________

(ii) Kindly mention any other competencies you may like to include for this EPA.

____________________________________________

- Any suggestions to improve/rephrase EPA.

_____________________________________

(ii) Kindly mention any other competencies you may like to include for this EPA.

____________________________________________

- Any suggestions to improve/rephrase EPA.

____________________________________________

(ii) Kindly mention any other competencies you may like to include for this EPA.

____________________________________________

Q1. What assessment strategies (formative and summative) should be employed for the following EPAs? You can choose more than one method.

Q2. What level of supervision should be employed for the following EPAs? Please choose only one level.

  Level 1 = Learner is allowed to be present and observe, not to undertake an EPA.
  Level 2 = Learner is allowed to execute EPA with direct, active supervision.
  Level 3 = Learner is allowed to carry out EPA without a supervisor in the room, but available if needed (indirect, reactive supervision).
  Level 4 = Learner is allowed to work unsupervised.
  Level 5 = Learner is allowed to provide supervision to more junior learners.
